# The Full-Length Transcriptome Sequencing and Identification of Na^+^/H^+^ Antiporter Genes in Halophyte *Nitraria tangutorum* Bobrov

**DOI:** 10.3390/genes12060836

**Published:** 2021-05-28

**Authors:** Liming Zhu, Lu Lu, Liming Yang, Zhaodong Hao, Jinhui Chen, Tielong Cheng

**Affiliations:** 1 Key Laboratory of Forest Genetics & Biotechnology of Ministry of Education of China, Co-Innovation Center for Sustainable Forestry in Southern China, Nanjing Forestry University, Nanjing 210037, China; zhulm20160918@163.com (L.Z.); lulu2020@njfu.edu.cn (L.L.); haozd@njfu.edu.cn (Z.H.); chenjh@njfu.edu.cn (J.C.); 2 College of Biology and the Environment, Nanjing Forestry University, Nanjing 210037, China; yangliming@njfu.edu.cn

**Keywords:** full-length transcriptome, SMRT, *Nitraria tangutorum*, *NtNHXs*

## Abstract

*Nitraria tangutorum* Bobrov is a halophyte that is resistant to salt and alkali and is widely distributed in northwestern China. However, its genome has not been sequenced, thereby limiting studies on this particular species. For species without a reference genome, the full-length transcriptome is a convenient and rapid way to obtain reference gene information. To better study *N. tangutorum*, we used PacBio single-molecule real-time technology to perform full-length transcriptome analysis of this halophyte. In this study, a total of 21.83 Gb of data were obtained, and 198,300 transcripts, 51,875 SSRs (simple sequence repeats), 55,574 CDS (coding sequence), and 74,913 lncRNAs (long non-coding RNA) were identified. In addition, using this full-length transcriptome, we identified the key Na^+^/H^+^ antiporter (*NHX*) genes that maintain ion balance in plants and found that these are induced to express under salt stress. The results indicate that the full-length transcriptome of *N. tangutorum* can be used as a database and be utilized in elucidating the salt tolerance mechanism of *N. tangutorum*.

## 1. Introduction

Nitraria is a type of typical sand shrub in the Zygophyllaceae [1,2]. *Nitraria tangutorum* is one of the species endemically distributed in the northwestern region of China [3,4]. *N. tangutorum* is one of the pioneer species that is resistant to salt, alkali, and sand burial, thereby playing an important role in maintaining the ecological balance of northwestern China [3,5]. In addition, this species has both economic value and benefits. Its leaves can be consumed, and its fruits can be used as food and medicine [6,7].

However, the current research on the omics of *N. tangutorum* is relatively scarce. In recent years, omics has rapidly developed into a powerful tool for studying various biological processes. Genomics, transcriptomics, and other omics can provide a large amount of genetic information [8], which may be utilized to conduct comprehensive studies on *N. tangutorum*.

The transcriptome is the sum of all mRNAs in a cell, tissue, or organism in a specific environment [9]. Transcriptome sequencing is helpful in the discovery of potential genes and can obtain a large number of gene sequences for molecular studies. The full-length transcriptome includes full-length mRNA sequences and complete transcripts with complete structural information [10]. Due to the limitation of reading length, second-generation sequencing technology can only obtain transcriptomes by splicing. Single-molecular real-time technology (SMRT) based on third-generation sequencing has obvious advantages in the reading length of sequencing [11,12]. It does not need the splicing of transcripts to obtain the full-length transcripts directly, which can more truly reflect the sequence information of a species and also help in the accurate analysis of the expression of homologous genes and family genes.

Whole-genome sequencing can provide extremely rich genetic information [13]. However, only 548 plant genomes were sequenced before 8 March 2021 (https://www.plabipd.de/index.ep (accessed on 25 May 2021)). Species that do not have completed whole-genome sequencing often have limited genetic information that hinders further research. Full-length transcriptome sequencing can provide extensive genetic information that can be used as a database to provide support for molecular and gene function research [14].

In this study, we used PacBio SMRT sequencing technology to obtain the full-length transcriptome of *N. tangutorum*, which was used in bioinformatics analysis, including full-length transcriptome correction and analysis, CDS prediction transcription factor analysis, SSR analysis, and lncRNA analysis, followed by functional annotation of the obtained transcripts based on the NR, Swiss-Prot, GO, COG, KOG, and KEGG databases. At the same time, we analyzed the full-length transcriptome, identified Na^+^/H^+^ antiporter genes, and verified their expression patterns under salt stress. These studies lay the foundation for elucidating the salt tolerance mechanism of *N. tangutorum* using molecular and omics technologies.

## 2. Materials and Methods

### 2.1. Plant Materials

*N. tangutorum* seeds were collected from Dengkou County, Inner Mongolia. The seeds were mixed in sand at 4 °C for about two months and then were planted in a mixed soil matrix with peat soil: perlite at a ratio of 4:1 in a greenhouse with a 16 h-light/8 h-dark light cycle and 60% air humidity at 23 °C. When the seedlings were 2 months old, the roots, stems, and leaves of three individual seedlings showing similar growth rates were collected and stored at −80 °C for RNA extraction.

### 2.2. RNA Extraction, Library Construction and SMRT Sequencing

Total RNA was extracted using an RNA extract kit (RNAiso Plus Co 9108, Takara, Japan), the qualified RNA of three seedings were mixed together, then used oligo dT as primer for cDNA synthesis using PrimeScript 1st Strand cDNA Synthesis Kit (Takara, Shiga, Japan) and amplified by PCR, then the cDNA fragments were screened by a BluePippin nucleic acid recovery system (Sage Science, APG BIO, USA). Then, the full-length cDNA is nicked and repaired, end-repaired, and SMRT adapters were connected to complete the library construction. Then, Agilent 2100 was employed to detect the insert size of the library, qRT-PCR was performed to accurately quantify the library and then sequenced using SMRT technology.

### 2.3. SSR Analysis

For SSR analysis, we screened unigene sequences of > 1000 bp in length. The MISA software (http://pgrc.ipk-gatersleben.de/misa/ (accessed on 25 May 2021)) was used to identify six types of SSRs, namely, mononucleotides, dinucleotides, trinucleotides, tetranucleotides, pentanucleotides, and hexanucleotides.

### 2.4. CDS, TF, and lncRNA Analysis

TransDecoder software was used to identify CDSs, iTAK software was used to predict TFs (transcription factors), and CNCI (Coding-Non-Coding Index) tools (https://github.com/www-bioinfo-org/CNCI (accessed on 25 May 2021)), CPC (Coding Potential Calculator) software (http://cpc2.gao-lab.org/ (accessed on 25 May 2021)), and Pfam (protein family) were employed to predict the coding potential of sequencing data.

### 2.5. Gene Functional Annotation

BLAST was used to compare unigene sequence with those in the NR (Non-Redundant protein sequence database, ftp://ftp.ncbi.nih.gov/blast/db (accessed on 25 May 2021)), Swiss-Prot (Swiss-Prot protein sequence database, http://www.uniprot.org (accessed on 25 May 2021)), GO (Gene Ontology Consortium, http://www.geneontology.org (accessed on 25 May 2021)), COG (Cluster of Orthologous Groups of proteins, http://www.ncbi.nlm.nih.gov/COG (accessed on 25 May 2021)), KOG (euKaryotic Orthologous Groups, http://www.ncbi.nlm.nih.gov/KOG (accessed on 25 May 2021)), and KEGG (Kyoto Encyclopedia of Genes and Genomes, http://www.genome.jp/kegg/ (accessed on 25 May 2021)) databases to obtain unigene annotation information.

### 2.6. Identification and Multi-Segment Alignments of Na^+^/H^+^ Antiporter Genes

Using the spliced sequence as the library, the BLAST program and Hmmer (v.3.0.1b) were used to search for genes containing the Na^+^/H^+^ antiporter domain (PF00999) with an E-value of <1 × 10^−5^, and then the SMAT online tool (http://smart.embl-heidelberg.de (accessed on 25 May 2021)) was employed to identify genes containing the domain. Multiple alignments were performed using DNAMAN v9.0 (Lynnon Corporation, San Ramon, CA, USA) with the deduced amino acid sequences.

### 2.7. Phylogenetic and Motif Analyses

Phylogenetic reconstruction was performed using MEGA-X v10.1 (Temple, Philadelphia, PA, USA) using the neighbor-joining method, and bootstrap values were calculated according to 1000 repetitions. For motif analysis, the online analysis software MEME (https://meme-suite.org/meme/tools/ (accessed on 25 May 2021)) was used to analyze protein conserved motifs, and the Tbtools [15] were used for drawing.

### 2.8. Gene Cloning

Total RNA was extracted from *N. tangutorum* seedlings using RNA extraction kit (Eastep super total RNA extraction kit Co LS1040, Promega, China), and the first strand of the cDNA was synthesized using HiScript III 1st Strand cDNA Synthesis Kit (Vazyme, China). The specific primers used to amplify the target gene fragments were designed with Oligo 7.60 (Cascade, Co 80809, USA) and are shown in Supplementary Table S7.

### 2.9. QRT-PCR Analysis

RNA was extracted from *N. tangutorum* seedlings exposed to 500 mM NaCl for 1, 6, 12, 24, and 48 h and then reverse transcribed for quantitative real-time-PCR (qRT-PCR). qRT-PCR was performed using a SYBR mix (AceQ qPCR SYBR Green Master Mix Co Q121-02, Vazyme, China). The PCR conditions were 94 °C for 15 s, 60 °C for 30 s, and 72 °C for 30 s. The primers used are shown in Supplementary Table S8, and each reaction was performed in triplicate. Relative expression values were calculated using the relative quantization method (2^−ΔΔCT^).

### 2.10. Subcellular Localization

ProtComp 9.0 (http://linux1.softberry.com/berry.phtml (accessed on 25 May 2021)) was employed for subcellular location prediction, and TMHMM (http://www.cbs.dtu.dk/services/TMHMM/ (accessed on 25 May 2021)) was utilized for protein transmembrane analysis. For gene subcellular localization, *pJIT-166* was used as the target vector, and *Xba*I and *Bam*HI were employed to generate restriction sites to construct a fusion expression vector. The primers used are shown in Supplementary Table S9. The expression vector was transfected into onion epidermal cells by microprojectile bombardment and incubated for 24 h in the dark [16]. The cells were subsequently observed using a fluorescence microscope (X-cite 120Q, Carl Zeiss).

## 3. Results

### 3.1. Sequencing Data Statistics

**Figure 1:**
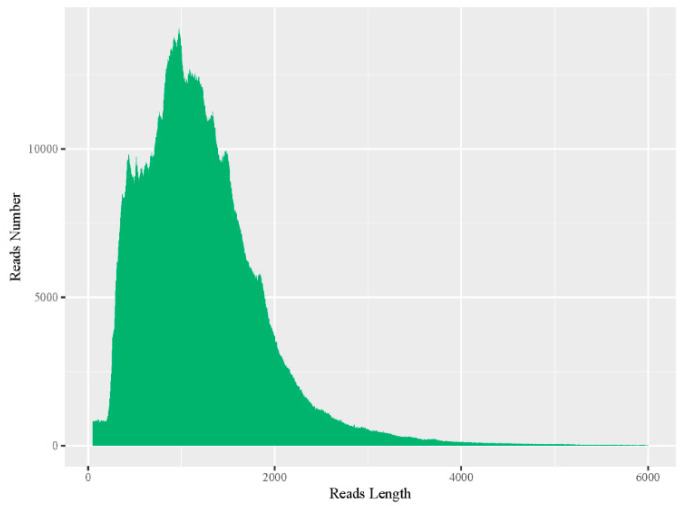
Distribution of subread length. The abscissa is the length of the subreads, and the ordinate is the number of subreads of a particular length.

Total RNA was extracted from the roots, stems, and leaves of *N. tangutorum* to construct a sequencing library, and SMRT was employed to sequence the qualified library, removing errors and redundant consensus sequences in the original sequence and the obtained data statistics are shown in Table 1. A total of 21.83 Gb raw data were obtained, representing a total of 17,951,056 subreads. Most of the lengths were within the size range of 1 to 2000 bp (Figure 1), the average length was 1216 bp, and the assembled N50 reached 1427 bp.

### 3.2. Data Correction

**Figure 2:**
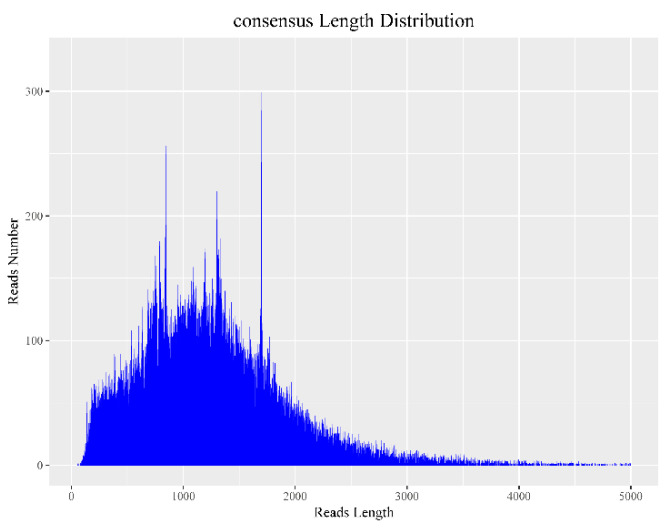
Consensus length distribution. The abscissa is the length of consensus, and the ordinate is the number of the consensus length.

CCS sequencing analysis can effectively reduce the error rate of sequencing to obtain more accurate data [17]. To further correct the accurate full-length transcriptome data, we analyzed the statistical results of the consensus sequence distribution in Table 2. At the same time, we also calculated the consensus length and constructed a consensus length distribution map and found that the main length of the consensus sequence was 1–2000 bp (Figure 2).

### 3.3. CDS, TFs, lncRNA Analysis

**Figure 3:**
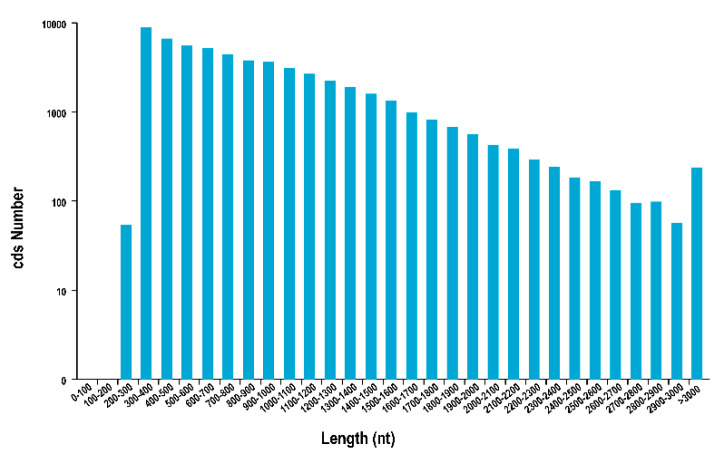
Coding sequence length distribution. The abscissa is the predicted length of CDSs, the left ordinate is the number of transcripts of the CDSs, and the right ordinate is the percentage of transcripts.

**Figure 4:**
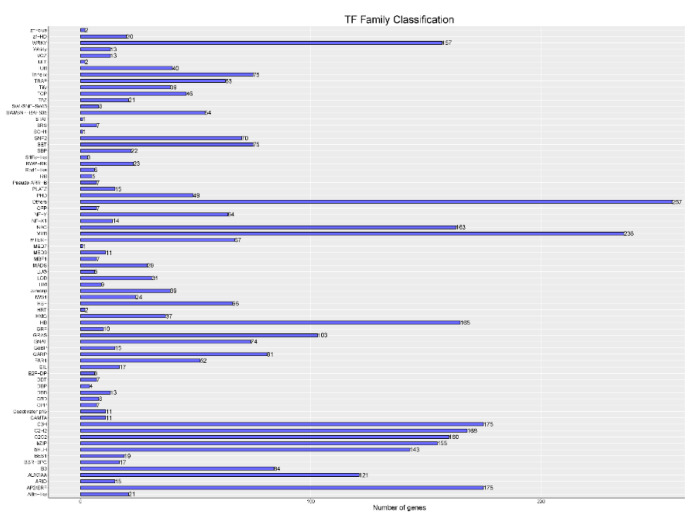
Transcription factor analysis. The ordinate shows different transcription factor families, and the abscissa depicts the number of the transcription factor families.

**Figure 5:**
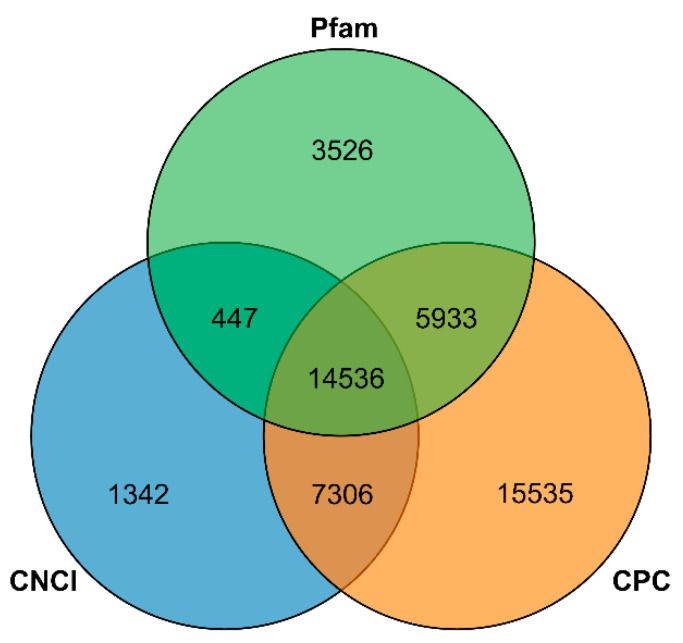
Coding ability prediction of Venn diagram. The blue circle, green circle, and yellow circle represent CNCI, CPC, Pfam, respectively.

Using TransDecoder software sequencing data to perform coding sequence prediction, a total of 55,574 sequences were obtained. The length distribution of the sequences is shown in Figure 3, and the distribution frequency is shown in Table S1. Using iTAK software, a total of 3745 transcription factors were predicted that belonged to 75 gene families (Figure 4), including *WRKY* (157), *MYB* (236), *NAC*, *AP2/ERF* (175), *bZIP* (155), and other transcription factors. CNCI, CPC software, and Pfam database were used to predict the coding potential of the sequencing data. CNCI analysis identified 74,913 lncRNAs (Table S2). The CPC software was used to classify and identify lncRNA. A total of 139,217 lncRNAs were identified (Table S3). Using the Pfam database to perform multiple sequence alignments and HMMs searches on PacBio sequencing data, a total of 90,061 lncRNAs were identified (Table S4). Finally, a Venn diagram was constructed to depict the lncRNAs identified by the three methods to identify shared sequences (Figure 5).

### 3.4. SSR Analysis

**Figure 6:**
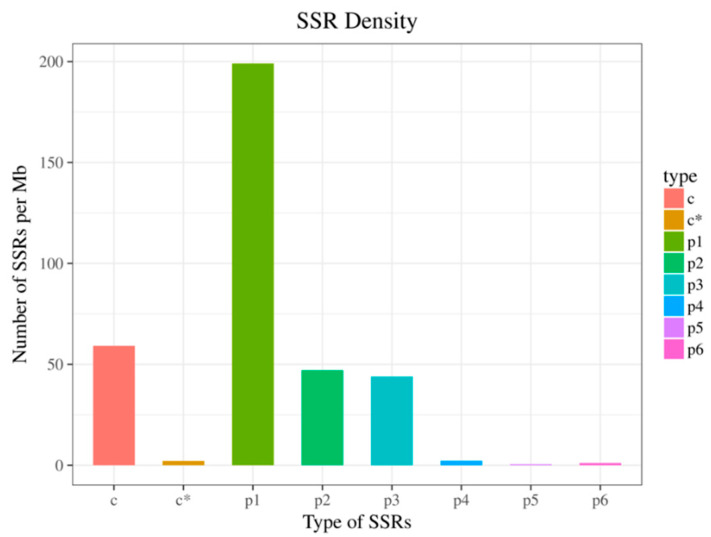
SSR density distribution. The abscissa is the SSR type, and the ordinate is the number of SSRs of the corresponding type in each Mb of sequence.

Using MISA software to perform SSR analysis on unigenes of >1.000 bp in length, a total of 51,875 SSRs were identified, most of which were mononucleotides, dinucleotides, and trinucleotides, accounting for 71.68%, 15.04%, and 12.13% of the total number of SSRs, respectively (Table 3), whereas tetranucleotides, pentanucleotides, and hexanucleotides collectively accounted for 11.47% of the total number of SSRs. At the same time, SSR density analysis also generated similar results (Figure 6).

### 3.5. Functional Annotation

The 65,361 unigenes were functionally annotated using the COG, GO, KEGG, NR, Swiss-Prot, and KOG databases (Table 4), with annotation coverage rates of 34.46%, 69.19%, 31.43%, 99.53%, 78.99%, and 61.89%, respectively.

**Figure 7:**
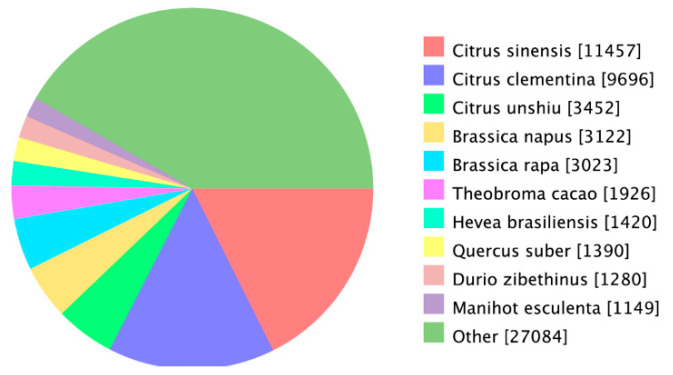
Homologous species distribution by Nr database. The best hits with an *E*-value = 1.0 × 10^−5^ for each query were grouped according to species.

**Figure 8:**
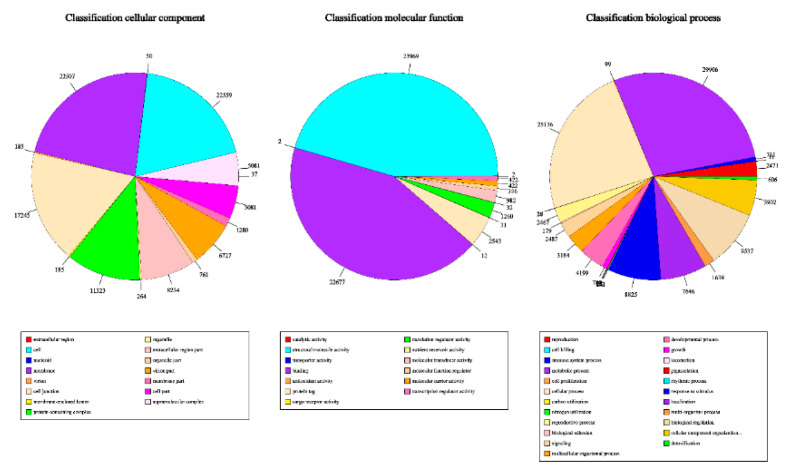
GO function classification.

Unigene annotation using the NR database revealed that these exhibited a high degree of homology with Citrus sinensis and C. clementina **(**Figure 7). We compared the annotated genes to the GO database and divided them into three independent classifications: cellular component, molecular function, and biological process, respectively (Figure 8).

**Figure 9:**
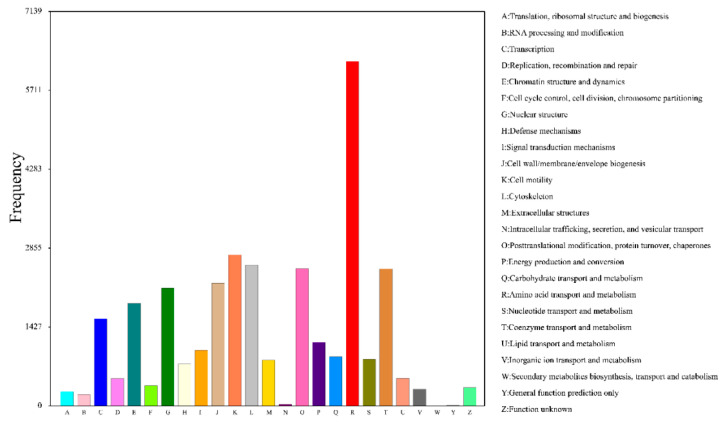
COG annotation classification statistics of expressed genes.

**Figure 10:**
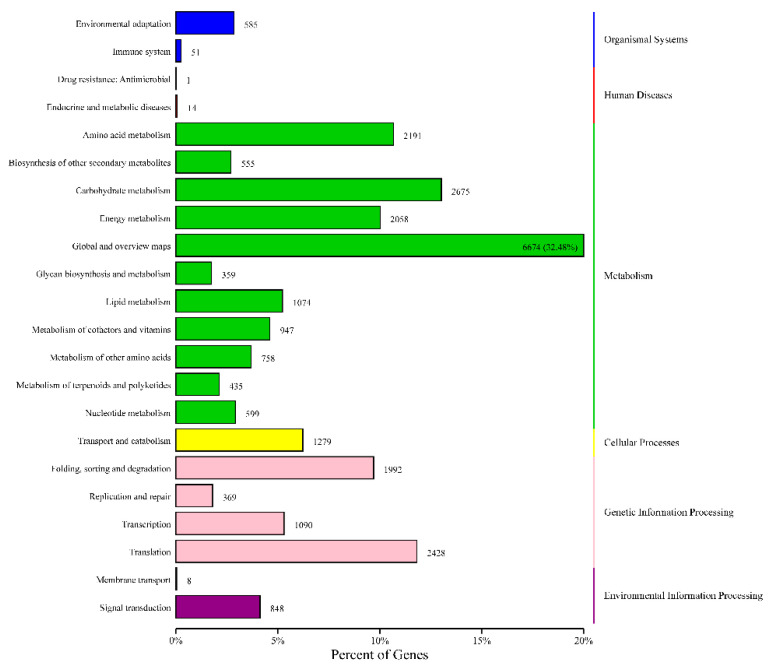
KEGG metabolic categories in the transcriptome of Nitraria tangutorum. The ordinate is the annotated biological function, and the abscissa is the percentage of these genes in the total number of annotated genes.

COG analysis classified these unigenes into 25 categories. Amino acid transport and metabolism was the most frequent functional category, followed by cell motility (Figure 9). KEGG annotation annotated 20,548 unigenes to 150 pathways (Table S5), which can be divided into 22 categories (Figure 10).

### 3.6. Identification of Na^+^/H^+^ Antiporter Genes in the Full-Length Transcriptome

**Figure 11:**
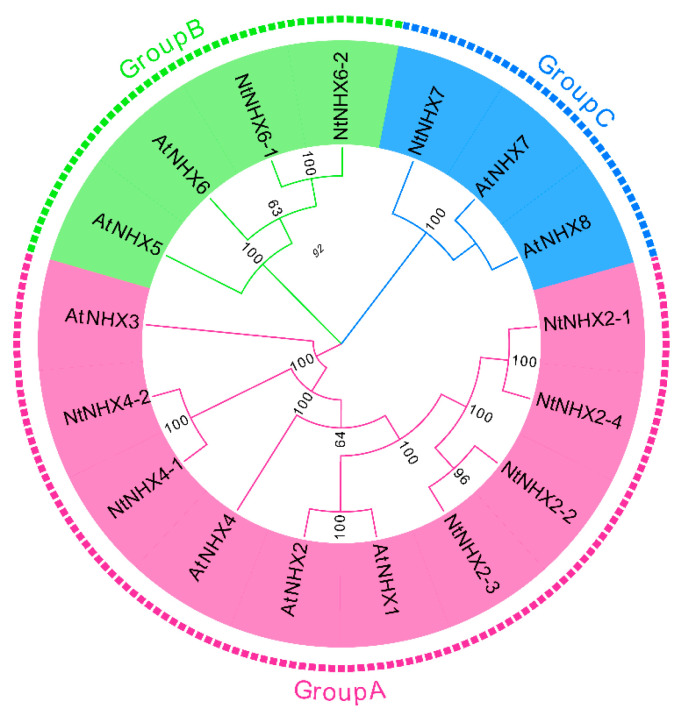
Phylogenetic analysis of NHX genes in *Nitraria tangutorum* and *Arabidopsis thaliana* (Linn.) Heynh.

The full-length transcriptome has a resolution accuracy of a single nucleotide, thus allowing the discovery of gene transcripts. N. tangutorum is a typical halophyte, but its molecular mechanism for salt tolerance remains unclear. The NHX gene family plays an important role in stress resistance in plants. However, its distribution in N. tangutorum is unknown. Using the full-length transcriptome as a database, we searched for sequences containing the domain according to the Na^+^/H^+^ antiporter (PF00999) Pfam domain and manually compared it to SMAT online analysis for confirmation. Using these steps, we have identified a total of nine NtNHXs, and then we named these based on their homology to AtNHXs. Multiple sequence alignment shows that these all contain a Na^+^/H^+^ antiporter domain (Supplemental Figure S2), then phylogenetic reconstruction of the AtNHXs was performed, which showed that these genes could be divided into three groups (Figure 11).

### 3.7. Gene Conserved Motifs Analysis of NHXs

**Figure 12:**
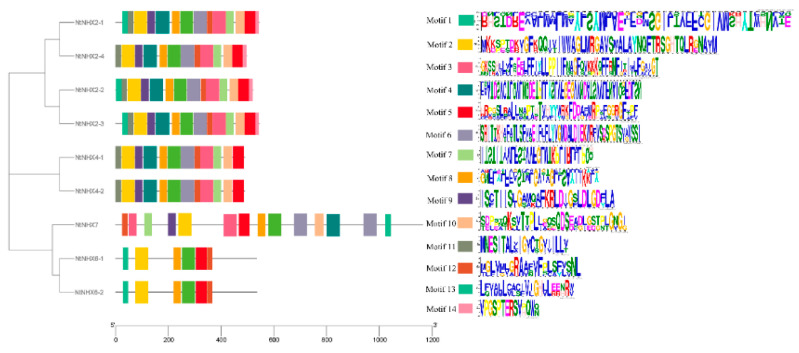
Conserved motif analysis of NtNHXs.

To identify the conserved motifs of these NtNHXs proteins, using MEME software, we determined that NtNHXs had 14 mainly conserved motifs (Figure 12). Almost all identified NtNHXs harbored similar motif sequences, indicating that these motifs were highly conserved.

### 3.8. Real-Time Quantitative PCR Analysis

**Figure 13:**
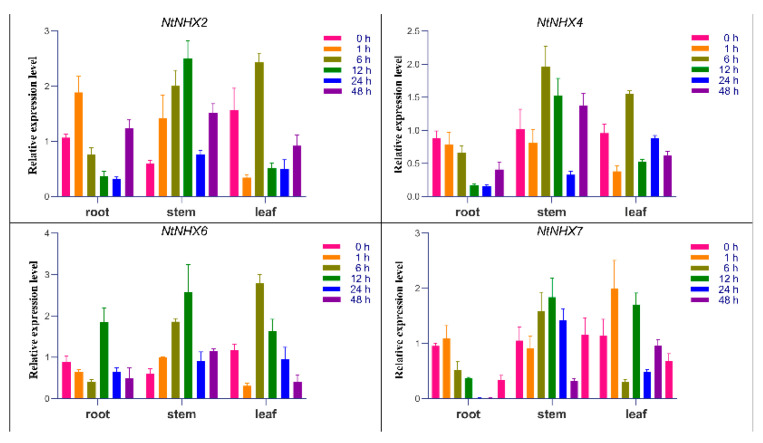
Relative expression level of NtNHXs in Nitraria tangutorum under 500 mM NaCl salt stress.

NHXs play an important role in maintaining the balance of Na^+^/H^+^ and salt resistance in plants [18,19]. To verify whether these NtNHX genes play a similar role in the salt-tolerance process of N. tangutorum, the seedlings of N. tangutorum were subjected to stress using 500 mM NaCl solution and plant materials were collected at 1, 6, 12, 24, and 48 h to assess NtNHX expression. Figure 13 shows that the NtNHX genes were expressed under salt stress, indicating that NtNHXs may play an important role in the salt resistance of N. tangutorum.

### 3.9. Clone and Subcellular Localization of NtNHX7

In *Arabidopsis*, AtNHX7 is also known as AtSOS1, which encodes a Na^+^/H^+^ antiporter that is located in the plasma membrane of the cell that promotes Na^+^ efflux and maintains the balance of ions inside and outside the cell [20]. To explore whether NtNHX7 has a similar effect and also to verify the accuracy of the predicted gene, we designed specific primers and cloned NtNHX7. We compared the sequence difference between the cloned gene and the reference sequence from this transcriptome, and we found that their similarity is 99.8% (Figure S1), indicating that the NtNHX7 predicted by the full-length transcriptome has higher accuracy.

**Figure 14:**
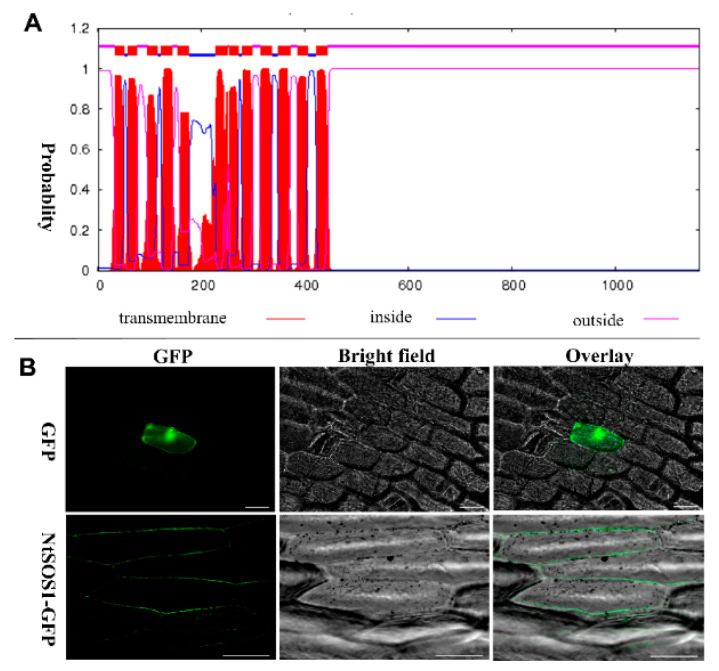
Subcellular localization of NtNHX7. (**A**) NtNHX7 transmembrane helix domain prediction by TMHMM. (**B**) NtNHX7 subcellular localization by microprojectile bombardmen, 35S: GFP plants as control. Scale bar =100 μm.

Real-time quantitative PCR analysis revealed that NtNHX7 is expressed by salt stress (Figure 13). Subsequently, we used the sequencing results to analyze its expression pattern. First, ProtComp 9.0 and TMHMM were used to predict the subcellular location pattern of NtNHX7, which showed that NtNHX7 has a higher probability of localizing to the plasma membrane of the cell (Table S6, Figure 14A). To further determine the subcellular localization of NtNHX7, we constructed the NtNHX7-GFP vector and performed transient transformation experiments to confirm our findings. As shown in Figure 14B, NtNHX7 is mainly expressed on the cell membrane, which also verifies our previous bioinformatics prediction.

## 4. Discussion

The SMRT technology is one of the relatively fast-developing bioinformatics technologies in recent years [21]. It can be used to improve the gene annotation status of sequenced species and as a reference for unsequenced species to facilitate scientific research. For example, the annotation of the sorghum genome has been improved using the SMRT technology, which will help to further study sorghum [22].

In sugar beet, the SMRT technology has been used to predict new genes and has laid the foundation for further research on this species [23].

Currently, transcriptome sequencing is mainly performed through NGS sequencing [24]. However, due to the limitation of sequencing read length in traditional next-generation sequencing, transcript information is mostly obtained by splicing sequences, and the full-length transcript sequence cannot be directly obtained. This hampers the analysis of transcriptome structure and may be accompanied by a high frequency of false-positive sequences.

Compared with second-generation sequencing, third-generation sequencing has a significantly higher read length, reaching an average of > 10 kb, and the maximum can reach 60 kb [25]. The SMRT technology can obtain a complete transcript without splicing, which is of great significance for transcriptome structure analysis, prediction of new genes [26], and supplementary genome annotation [27].

At present, various species have completed whole-genome sequencing, but there are still numerous organisms that have not completed the construction of a whole-genome map due to limited attention or sequencing cost, which also hinders further research on these species. For species that have not completed whole-genome sequencing, the full-length transcriptome provides a wealth of genetic information. Transcriptome data can be used to clone genes, develop SSR primers [28], and analyze homologous genes. 

*N. tangutorum* is a typical drought-tolerant and salt-tolerant plant that is of important ecological significance. However, whole-genome sequencing of this species has not been completed to date, and limited genetic information hinders related molecular research. Although some genes of *N. tangutorum* have been cloned and their functions have been verified, this is only the tip of the iceberg in terms of studying this species from a molecular perspective. Using its full-length transcriptome sequencing data, it is possible to analyze its CDS, transcription factors, and SSRs and at the same time perform functional annotations on its genes and develop SSRs primers for plant population classification [29].

Full-length transcriptomes can also be used as a database to identify target genes [30]. *Nitraria tangutorum* is a typical halophyte, and we would like to study the expression of salt-resistance-related genes in it. In the study of other plants such as *Arabidopsis* [31] and rice [18], *NHXs* have been shown to be involved in the process of salt stress tolerance. Therefore, in this study, we used the full-length transcriptome data as a library to obtain sequence information on the *NtNHXs* using BLAST. Then we investigated whether these genes responded to salt stress, and we found they can be induced by salt stress, although they had different expression characteristics in different tissues after salt treatment. In general, their expression reached its maximum within 12 h and then decreased (Figure 13), which was similar to the study of *Gossypium barbadense* [32] and *Pyrus betulaefolia* [33], but there were also some differences, we suspect that the reason for this difference might be related to differences in salt stress concentration and species. In summary, the gene expression results indicated that the *NtNHX* gene might be involved in the process of resisting salt stress in *Nitraria tangutorum*, but further gene functional verification studies are needed.

In addition, we selected *NtNHX7* to be cloned and sequenced and found that the cloned gene sequence was basically consistent with the sequence provided by the full-length transcriptome (Figure S1) through sequencing. Further, we constructed the NtNHX7-GFP gene fusion expression vector and observed its expression pattern through microprojectile bombardment and found that the signal was mainly expressed on the plasma membrane and distributed inhomogeneously (Figure 14), which was similar to the expression pattern of the *AtNHX7* (also known as *AtSOS1*) in *Arabidopsis* [20]. 

According to the above results, we thought that the full-length transcriptome could be used as a gene database, although it may not be as complete when compared with the full-genome sequence. However, for unsequenced species, it can enrich the scarce omics information and also lay the foundation for further molecular research.

## 5. Conclusions

In this study, we used SMRT technology to determine the full-length transcriptome of *N. tangutorum*. A total of 21.83 Gb of data were obtained, of which 198,300 transcripts, 51,875 SSRs, 55,574 CDS, and 74,913 IncRNAs were identified. In addition, using this full-length transcriptome, we identified the key *NHX* genes that maintain ionic balance in plants, and we induced their expression under salt stress. Based on the full-length transcriptome data, we also cloned the *NtNHX7* gene and found a high similarity to the predicted result through sequencing, which indicates that the gene prediction based on the full-length transcript is more accurate.

In summary, this study obtained high-quality, full-length transcript information on *N. tangutorum* by full-length transcription sequencing and provided abundant transcript information, which promotes omics and molecular research on *N. tangutorum*.

## Figures and Tables

**Table 1:** Full-length transcriptome sequencing data.

Sample	Subreads Base (G)	Subreads Number	Average Length of Subreads	N30	N50	N90
*N. tangutorum*	21.83	17,951, 056	1216	1788	1427	744

**Table 2:** CCS quantity statistics data.

Sample	CCS	Nfl-Reads	Flnc-Reads	Mean-Flnc	Consensus Reads
*N. tangutorum*	21.83	179,510, 56	1216	1788	1427

CCS (circular consensus sequence), flnc-reads (full-length non-chimeric reads), Mean-flnc (average length of full-length non-chimeric reads), Consensus-reads (reads of consensus sequence obtained after clustering).

**Table 3:** Results of SSR analysis.

Feature	Number
Total number of sequences examined	71,089
Total size of examined sequences (bp)	111,431,918
Identified SSRs	51,875
SSR containing sequences	29,249
Sequences containing more than 1 SSR	11,082
SSRs present in compound formation	12,283
Mononucleotides	37,182
Dinucleotides	7804
Trinucleotides	6294
Tetranucleotides	330
Pentanucleotides	89
Hexanucleotides	176

**Table 4:** Unigene annotation statistics.

Annotated Databases	Number of Unigenes	300–1000 bp	≥1000 bp
COG	26,526	3596	22,903
GO	45,222	7626	37,501
KEGG	20,548	3528	16,970
KOG	40,450	6479	33,887
Pfam	47,073	6746	40,303
Swiss-Prot	51,635	8760	42,752
Nr	65,055	12,596	52,211
All	65,361	12,728	52,376

The column header 300–1000 bp indicates the number of unigenes annotated to the database whose length is ≥300 bases and ≤1000 bases. The column header ≥ 1000 bp indicates the number of unigenes annotated to the database unigene number with lengths ≥ 1000 bases.

## Data Availability

All data generated or analysed during this study are included in this published article and its Supplementary Information Files. Raw data generated for this study have been uploaded to NCBI with accession number is SAMN19103071.

## Supplementary Materials

The following are available online at https://www.mdpi.com/article/10.3390/genes12060836/s1, Figure S1: The amino acid sequence comparison between predicted sequence and sequencing result of NtNHX7. The top row is the prediction result, and the second row is the sequencing result. Figure S2: The amino acid multiple fragment alignments of NtNHXs. Table S1: CDS length distribution and its frequency. Table S2: CNCI analysis for lncRNAs. Table S3: CPC analysis for lncRNAs. Table S4: Pfam analysis for IncRNAs. Table S5: KEGG annotation databases. Table S6: Prediction of NtNHX7 subcellular localization by ProtComp9.0 (http://linux1.softberry.com/berry.phtml (accessed on 25 May 2021)). Table S7: Primers for gene cloning. Table S8: Primers for Quantitative RT-PCR. Table S9: Primers for subcellular localization. Table S10: amino acid sequence of NtNHXs.

## Abbreviations

NHX	Na^+^/H^+^ antiporter gene
SRMT	Single-molecule real-time
CDS	Coding sequence
SSR	Simple sequence repeat
LncRNA	Long non-coding RNA
CPC	Coding potential calculator
CNCI	Coding non-coding index
CPC	Coding potential calculator
mRNA	Messenger RNA
qRT-PCR	Quantitative real-time PCR
TF	Transcription factors
cDNA	Complementary DNA
Pfam	Protein family
NR	Non-redundant protein sequence database
Swiss-Prot	Swiss-Prot protein sequence database
GO	Gene Ontology Consortium
COG	Cluster of Orthologous Groups of proteins
KOG	EuKaryotic Orthologous Groups
KEGG	Kyoto Encyclopedia of Genes and Genomes
