# Supplementary material for: The Full-Length Transcriptome Sequencing and Identification of Na+/H+ Antiporter Genes in Halophyte Nitraria tangutorum Bobrov
Source: Genes (Basel). 2021 May 28;12(6):836. doi: 10.3390/genes12060836 (PMC8227117; doi:10.3390/genes12060836)
Supplement: Supplementary file 1 [file genes-12-00836-s001.zip › genes-1211273-supplementary.pdf]

Supplementary Materials:

Figure S1: title, Table S1: title, Video S1: title.

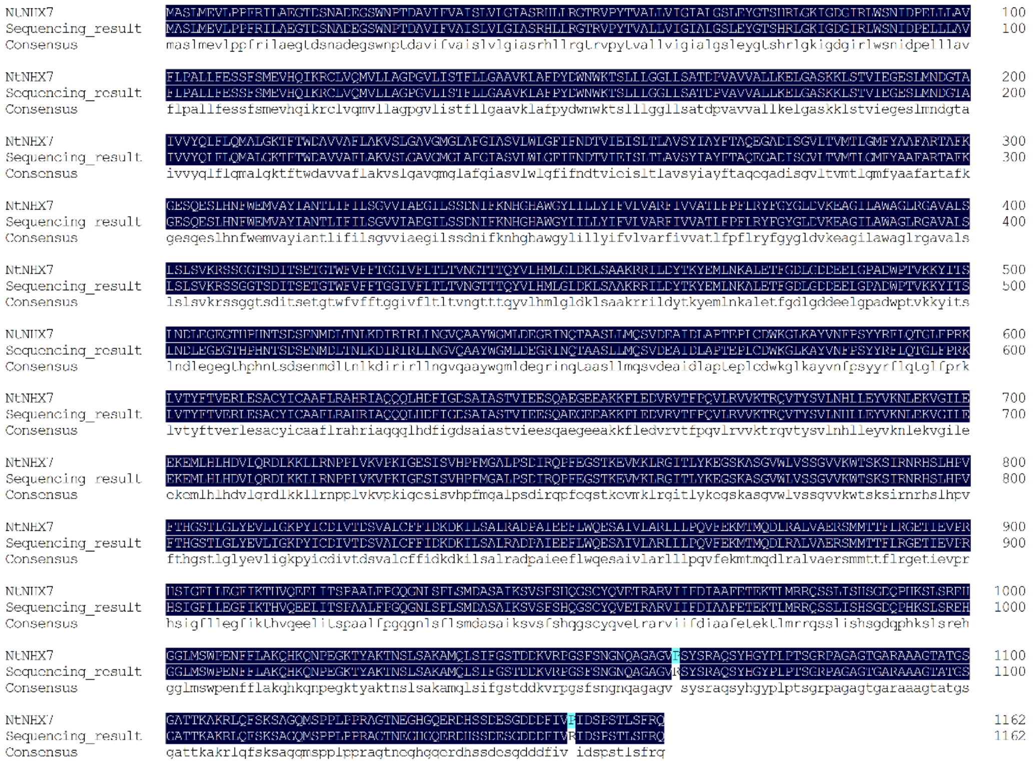

Figure S1: The amino acid sequence comparison between predicted sequence and sequencing result of NtNHX7. The top row is the prediction result, and the second row is the sequencing result.

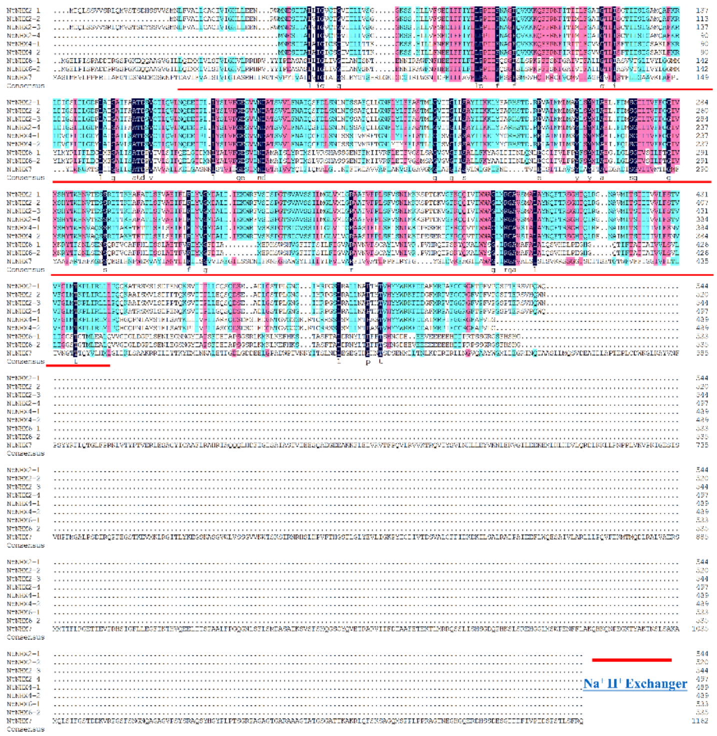

Figure S2: The amino acid multiple fragment alignments of NtNHXs
